# Pathogenic and Genotypic Characterization of a Japanese Encephalitis Virus Isolate Associated with Reproductive Failure in an Indian Pig Herd

**DOI:** 10.1371/journal.pone.0147611

**Published:** 2016-02-19

**Authors:** P. A. Desingu, Pradeep K. Ray, B. H. M. Patel, R. Singh, R. K. Singh, G Saikumar

**Affiliations:** 1 Swine Disease Lab, Division of Pathology, Indian Veterinary Research Institute, Izatnagar, Uttar Pradesh, India, 243122; 2 Division of Livestock Production and Management, Indian Veterinary Research Institute, Izatnagar, Uttar Pradesh, India, 243122; 3 Division of Pathology, Indian Veterinary Research Institute, Izatnagar, Uttar Pradesh, India, 243122; 4 Indian Veterinary Research Institute, Izatnagar, Uttar Pradesh, India, 243122; University of Missouri, UNITED STATES

## Abstract

**Background:**

India is endemic to Japanese encephalitis virus (JEV) and recurrent outbreaks occur mainly in rice growing areas. Pigs are considered to be the amplifying host for JEV and infection in gestating pigs results in reproductive failure. Most studies conducted on JEV infection in Indian pigs have been serological surveys and very little is known about JEV genotypes circulating in pigs. So the potential risk posed by pigs in JEV transmission and the genetic relationship between viruses circulating in pigs, mosquitoes and humans is poorly understood.

**Methodology/Principal Findings:**

This study was conducted in pigs with a history of reproductive failure characterized by stillborn piglets with neuropathological lesions. Japanese encephalitis (JE) suspected brain specimens inoculated intracerebrally into mice and Vero cells resulted in successful isolation of JEV/SW/IVRI/395A/2014. Clinicopathological observations in infected mice, demonstration of JEV antigen in brain, and analysis of the envelope protein identified the swine isolate as being neurovirulent. Phylogenetic analysis based on prM and E gene sequences showed that it belonged to genotype III. This swine isolate was closely related to JEV associated with the 2005 outbreak in India and JaoArS982 from Japan. Phylogenetic analysis of JEV strains collected between 1956 and 2014 in India categorized the GIII viruses into different clades blurring their spatial distribution, which has been discernible in the previous century.

**Conclusions/Significance:**

Isolation of JEV from stillborn piglets and its close genetic relationship with viruses detected at least three decades ago in humans and mosquitoes in Japan suggests that the virus may have been circulating among Indian pigs for several decades. The close similarity between the present swine isolate and those detected in humans affected in the 2005 outbreak in Uttar Pradesh, India, suggests the need for more intensive surveillance of pigs and implementation of suitable strategies to control JE in India.

## Introduction

Japanese encephalitis (JE) is an arthropod-borne zoonotic viral disease caused by Japanese encephalitis virus (*Flaviviridae*;*Flavivirus*; [JEV]). In the Australasian countries, where the disease is endemic, about 68,000 human JE cases and 10,000–15,000 deaths are estimated to occur every year [1–2]. In India, JE was first reported in 1955 from Vellore, in Tamil Nadu [3]. JE which was confined to south India spread to Burdwan and Bankura districts of West Bengal in 1973 and by 1978 it was recorded in 21 States of India [4–6]. Between 1978–2009, a dramatic increase in the number of JE cases was recorded in the Gorakhpur district of Uttar Pradesh [6].

The reliable diagnosis of JE is based on the isolation of the virus from2 to 4 day-old mice following intracerebral inoculation or on virus isolation from infected cell culture [7]. The neurovirulence of a JEV isolate is determined by intracerebral inoculation in 3–4 week old mice [8] and by molecular analysis of critical amino acid substitutions in the E protein [8–10]. Phylogenetic studies based on prM and E gene of JEV identified five different genotypes of JEV [10–11]. Genotype III is the predominant genotype found in the Indian subcontinent but the presence of genotype I in Gorakhpur region (Uttar Pradesh, India) in 2009 [12], and subsequently in 2010, in West Bengal [13] has also been reported.

Pigs act as amplifying hosts, and ardeid birds such as pond herons and egrets are the maintenance reservoirs for the virus. Mosquitoes, mainly Culex spp. act as vectors and human and equine are considered dead end hosts [7]. JEV infection in pregnant pigs can result in reproductive problems characterized by abortion, stillbirths and mummified fetuses [7, 14]. In India, extensive studies have been conducted on mosquitoes in JE endemic areas, and the virus has been recovered from 19 different mosquito species; but most studies on JEV infection in Indian pigs have been limited to seroprevalence in different parts of the country [15–16]. So far there is only one report of JEV isolation from a sentinel pig in India [17], but there is no information on the genotype involved and its genetic relationship with JEV strains detected in humans. This study was conducted in a pig herd in the State of Uttar Pradesh, which had a history of reproductive problems in pregnant sows. The pathological changes observed in stillborn piglets and the genetic characterization of the isolated virus are reported in this paper.

## Methods

### Ethics statement

The mice experiment in this study was conducted according to the guidelines of the Committee for the Purpose of Control and Supervision on Experiments on Animals (CPCSEA), India. Animals were handled in strict accordance with good animal practice, including humane endpoint treatment for mice, euthanasia, frequent monitoring of mice and reduced suffering and distress as defined by CPCSEA, Ministry of Environment and Forestry, Government of India. This study was approved by the Institute Animal Ethics Committee (IAEC), Indian Veterinary Research Institute (IVRI), Izatnagar, India [Permit number: F.1-53/2012-13/JD(R)]. The pig samples were drawn by the Swine Production Farm from naturally dead pigs submitted to the Post-Mortem Facility, Division of Pathology, IVRI, Izatnagar for disease investigation. The pig samples were drawn from naturally dead pigs (no experiment was carried out in live pigs), so ethical committee approval was not required.

### Study population

This study was conducted in a pig herd in Uttar Pradesh, India, from April 2014 to October 2014. Systematic necropsy was conducted on stillborn piglets and the gross lesions were recorded. Representative tissue samples were collected in 10% neutral buffer formalin (NBF) for histopathological processing. Tissue specimens collected on ice were stored under -20°C until further use for molecular and virus isolation studies.

### Histopathology and immunohistochemistry

The formalin-fixed tissues were processed for preparation of haematoxylin and eosin (H&E) stained slides for light microscopy following standard procedures.

For immunohistochemistry (IHC), 5 μm thick formalin-fixed paraffin-embedded tissue sections were cut and mounted on 3-Aminopropyltriethoxysilane (APES), (Sigma-Aldrich, USA)-coated slides and air dried for 30 min. Anti-JEV hyperimmune serum raised in rabbit (GeneTex, Irvine, CA, USA) was diluted to 1:150 in 1% BSA and Goat Anti-Rabbit IgG-HRPO conjugate (Genei, India) was used as the secondary antibody at 1:150 dilution in PBS. AEC (3-Amino-9-ethylcarbazole) (Sigma-Aldrich, USA) substrate was prepared as per manufacturer’s recommendation and counterstaining was done with Mayer’s hematoxylin. AEC stained sections were finally mounted with CC/Mount^™^ (Sigma-Aldrich, USA). For the negative control, primary antibody was substituted by 1% BSA and other procedures were followed as mentioned above.

### RNA extraction and RT-PCR

Viral RNA was extracted from brain samples using QIAamp Viral RNA Mini Kit (QIAGEN, Hilden, Germany) as per manufacturer’s instruction. The cDNA was synthesized using SuperScript^®^ III First-Strand Synthesis System (Invitrogen, Carlsbad, CA, USA). The RT-PCR for the detection of E and prM genes of JEV was performed following previously published protocols (Table 1) [18–19].

**Table 1:** Gene targets and nucleotide sequences of primers used to amplify JEV gene segments.

Gene	Primer	Sequence	Product size	Refs.
FLAVI NS5	FLAVI-1	5’-AATGTACGCTGATGACACAGCTGGCTGGGACAC- 3’	863 bp	Ayers et al., 2006
	FLAVI-2	5’-TCCAGACCTTCAGCATGTCTTCTGTTGTCATCCA–3’		
JEV-prM	JEV-prMf	5’-CGTTCTTCAAGTTTACAGCATTAGC-3’	675 bp	Wang et al., 2007
	JEV-prMr	5’-CCYRTGTTYCTGCCAAGCATCCAMCC-3’		
JEV-E	JEV-Ef	5’-TGYTGGTCGCTCCGGCTTA-3’	1582 bp	Wang et al., 2007
	JEV-Er	5’-AAGATGCCACTTCCACAYCTC-3’		

### Cloning and sequencing

RT-PCR amplicons were purified using GeneJET Gel Extraction Kit (Thermo Scientific, Waltham, Massachusetts, USA) and cloning of the amplified PCR products was carried using InsTAclone PCR Cloning Kit (Thermo Scientific, Waltham, Massachusetts, USA). The plasmids were extracted with GeneJET Plasmid Miniprep Kit (Thermo Scientific, Waltham, Massachusetts, USA) and positive clones were sequenced from both ends (Eurofins Genomics India Pvt. Ltd., Bangalore, India).

### Pathogenecity testing in mice

Mice for this study were obtained from the Laboratory Animal Resources (LAR) section, Indian Veterinary Research Institute (IVRI), Izatnagar, India. The inoculums were prepared as per OIE (7) recommendations (10% brain suspension) and 20 μl were inoculated intracerebrally into 3–4 week old mice (n = 6 mice). The mice were observed daily for neuro-pathological symptoms and sacrificed at 14 dpi (days post-infection). Systematic necropsy was conducted and samples were collected for histopathology, immunohistochemistry (IHC), virus isolation and molecular studies.

### Propagation of JEV in Vero cell culture

Vero cells were obtained from Division of Virology, Indian Veterinary Research Institute (IVRI), Mukteswar Campus, Nainital (Uttaranchal), India. For the isolation of JEV in Vero cells, brain samples (10% suspensions in phosphate-buffered saline) from mice showing neuro-pathological symptoms and samples from stillborn piglets were processed according to the OIE Terrestrial Manual [7]. In Vero cells, four passages were carried out and the cytopathic effects were noticed and the presence of JEV was confirmed by RT-PCR and IHC.

### Molecular analysis for neurovirulence of JEV

The critical amino acids in the E protein of JEV reported to be associated with neurovirulence [10] were analyzed by comparing the amino acid sequence of the present Indian isolate (JEV/SW/IVRI/395A/2014; from hereafter referred to as IVRI395A) with all available JEV (1956–2014) sequences (both GI and GIII viruses) from India and 150 viruses from other countries retrieved from the NCBI database.

### Molecular characterization of the E protein in Indian JEV from 1956–2014 at sites under positive selection

The amino acids in the E protein of JEV reported to be associated with positive selection were analyzed by comparing the amino sequence of the present Indian isolate (IVRI395A) with all available JEV (1956–2014) sequences (both GI and GIII viruses) from India retrieved from the NCBI database. The haplotypes based on the four sites in the E protein (E-123, E-209, E-227 and E-408) were analyzed [20].

### Phylogenetic analysis of JEV

Phylogenetic analysis of JEV isolates was performed for the E and prM genes Nucleotide sequences available for partial prM gene of Indian isolates of JEV (1956–2014) were used to construct separate phylogenetic trees for close comparison with the present swine isolate. Partial E gene sequences (296 nts) available for JEV isolates associated with the major outbreak of 2005 in the State of Uttar Pradesh, India, [21] and viruses from Japan (M18370, AB028257, U70414) and Indonesia (U70397, JN375549) were analyzed separately to establish their genetic relationship. All trees were constructed with MEGA6 software using neighbor-joining method based on the Kimura-2 parameter model, substitutions to include transition and transversion and uniform rate among sites. The codon positions 1st, 2nd, 3rd, and non-coding were included. All positions containing gaps and missing data were eliminated. To determine internal node and better differentiation of different clades within genotype III, cutoff value of 50 was used with bootstrap support values expressed as percentage for 1,000 replicates.

## Results

In total, 28 sows farrowed during the study period. Occurrence of stillbirth was recorded in 10 (7 crossbred and 3 Landrace) sows; with the number of stillborn piglets being 2 to 5 per sow (Table 2). Of 31 stillborn piglets (22 crossbred and 9 Landraces) delivered and tested by RT-PCR assay, seven (5 crossbred and 2 Landraces) piglets delivered by 2 sows were found positive for JEV.

**Table 2:** Sows which delivered JEV positive stillborn piglets in a herd. Out of 28 sows that farrowed in a herd, 10 sows delivered stillborn piglets. Sow number, date of farrowing, breed, number of stillbirth and RT-PCR results for JEV are presented.

S.No.	Sow No.	Date of Farrowing	CB/LR	No of Stillbirth	JEV RT-PCR
1	64	01.04.14	CB	4 (3M;1F)	N
2	768	23.04.14	LR	2 F	N
3	160A/14	24.04.14	CB	2 F	N
4	770	16.05.14	LR	5(4M; 1F)	N
5	116	23.05.14	CB	2M	N
6	754	29.05.14	LR	2(1M;1F)	P
7	27	01.06.14	CB	2M	N
8	146	05.06.14	CB	5 (3M;2F)	N
9.	115	08.09.14	CB	5 (3M; 2F)	P
10	66	08.09.14	CB	2F	N

CB- Crossbred; LR- Landrace; M-Male; F-Female; P- Positive; N-Negative

### Gross and microscopic lesions

**Fig 1:**
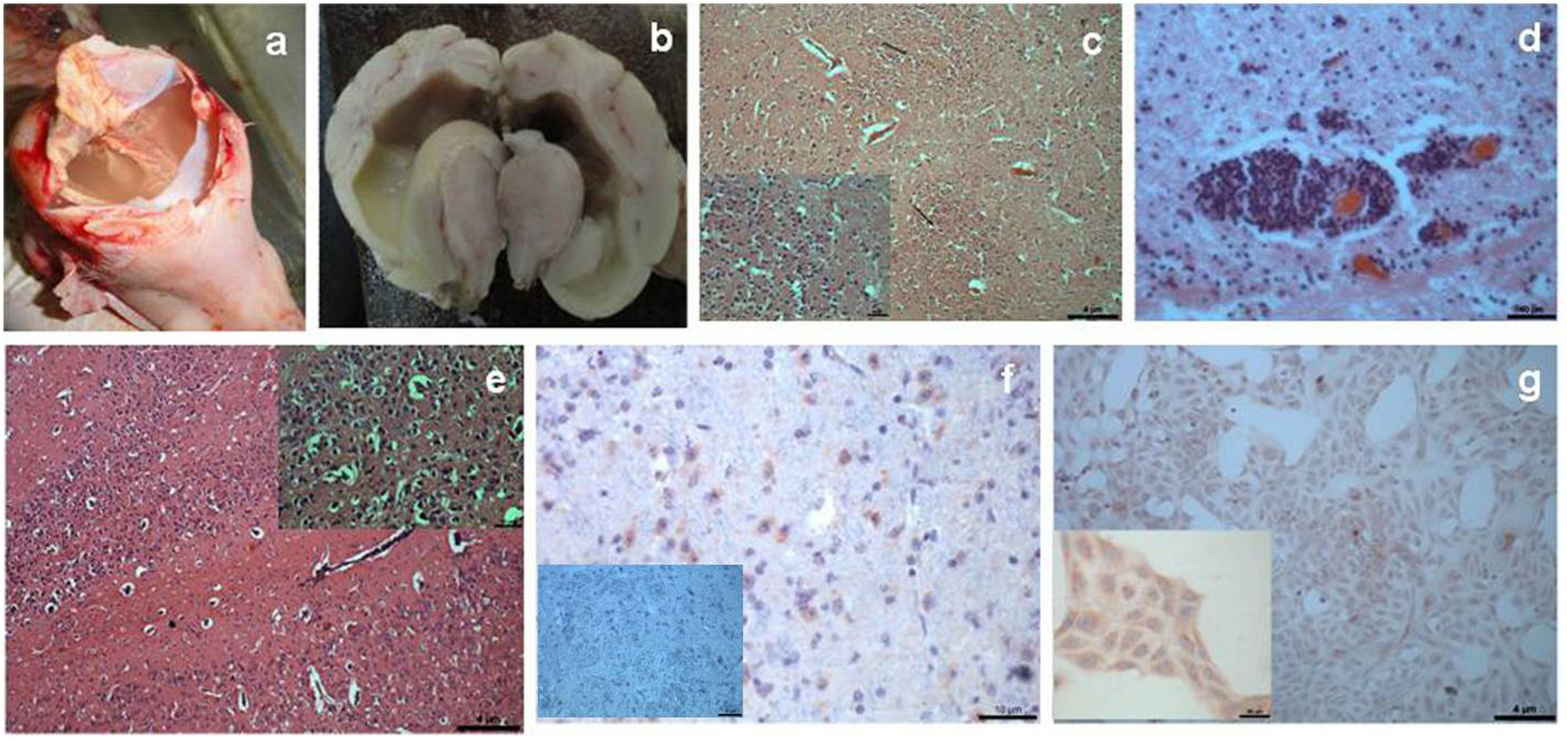
1a-JEV infected stillborn piglet with hydranencephaly; 1b- Cross section of a brain with severe dilatation of ventricular spaces and thinning of the surrounding parenchyma; 1c- Diffuse gliosis in the cerebrum of piglet, (insert higher magnification), H& E; 1d-Perivascular cuffing in the subependymal region of piglet brain, H& E; 1e- Severe neuronal degeneration and necrosis in the mice brain, (insert higher magnification), H& E; 1f- JEV antigen in the cytoplasm of cerebral neurons (rose red color), IHC; 1g- JEV antigen in the cytoplasm of Vero cells (rose red color), insert higher magnification, IHC.

Five stillborn piglets positive for JEV showed following pathological lesions in the brain. The head appeared more rounded and subcutaneous hemorrhages were observed over the head. Upon opening the skull, one piglet revealed hydranencephaly with grayish-white flaky fluid filling the entire cranial cavity and only remnants of brain parenchyma seen as small strands (Fig 1a). Other piglets had swollen, rounded brains with complete loss of sulci and gyri. Cross sections of these brains revealed severe dilatation of ventricular spaces and thinning of the surrounding parenchyma (Fig 1b). The carcass lymph nodes were prominently congested. The most significant microscopic lesions found in the brains were widespread edema, congestion and microhemorrhages in the parenchyma. Neuronal degeneration, and focal to diffuse gliosis (Fig 1c), occasional finding of perivascular cuffing around small vessels particularly in the vicinity of the ventricular spaces (Fig 1d) and accumulation of glial cells in the subependymal region were the prominent microscopic lesions. IHC demonstrated the presence of JEV antigen mainly in the cytoplasm of cerebral neurons.

### RT-PCR

JEV nucleic acids were detected in the brains of seven stillborn piglets from two crossbred sows in RT-PCR using three sets of primers (Table 1). In total, out of 33 stillborn piglets tested by RT-PCR, seven stillborn piglets from two sows were found positive for JEV.

### Pathogencity testing of JEV in mice

In mice (n = 6 mice) inoculated intracerebrally with brain suspensions from stillborn piglets neuro-pathological signs like reduced movement, circling and posterior paresis were observed between 11–14 dpi. At necropsy, no gross lesions were noticed. Microscopically, severe neuronal degeneration and necrosis, perivascular cuffing and gliosis were noticed (Fig 1e). IHC localized the JEV antigen in the cytoplasm of neurons in cerebrum (Fig 1f), cerebellum and midbrain.

### Propagation of JEV in Vero cell culture

The cytopathatic effect (rounding and detachment of cells) in infected Vero cell line was noticed at 5^th^ day of inoculation. Presence of JEV in infected Vero cells was confirmed by RT-PCR and IHC at 4^th^ passage (Fig 1g).

### Molecular analysis of neurovirulence

Neurovirulent specific critical amino acids in the E protein of IVRI395A and other Indian isolates from humans and mosquitoes are summarized in Table 3. The current swine isolate and all previous Indian isolates of JEV except KC526871 (human isolate from Malda, West Bengal, 2012) carried amino acids critical for neurovirulence.

**Table 3:** Comparative analysis of critical amino acids in the E protein associated with JEV neurovirulence. Eight amino acids in the E protein critical for neurovirulence in the vaccine strain (SA14-14-2) and other JEV strains including current swine isolate.

Amino acid position	Amino acids in		Swine isolate (JEV/SW/IVRI/395A/2014)	Other Indian isolates (81 G-III & 6 G-I)
	Vaccine Strain SA14-14-2	Neurovirulent Strains		
E-107	F	L	L	L
E-138	K	E	E	E
E-176	V	I	I	I, V (Z34097)
				T (AF080251; JX131374)
E-177	A	T	T	T
E-264	H	Q	Q	Q, H (KC526871)
E-279	M	K	K	K
E-315	V	A	A	A, V (KC526871)
E-439	R	K	K	K, R (KC526871)

### Molecular characterization of the E protein in Indian JEV from 1956–2014 at sites under positive selection

Five different haplotypes (E-123, E-209, E-227 and E-408) of JEV have been detected so far in India [20]. The predominant haplotype detected in humans and mosquitoes is SKSS. The present Indian swine isolate and all GI viruses reported from India belong to this haplotype. Four Indian JEV isolates, three from humans and one from mosquitoes, belong to the SKPS haplotype. The other three haplotypes, one each of RKSS, SRSS and SKTS, were detected in India. In comparison to other isolates, the E protein of IVRI395A had a unique amino acid substitution at E-169.

### Phylogenetic analysis of JEV

**Fig 2:**
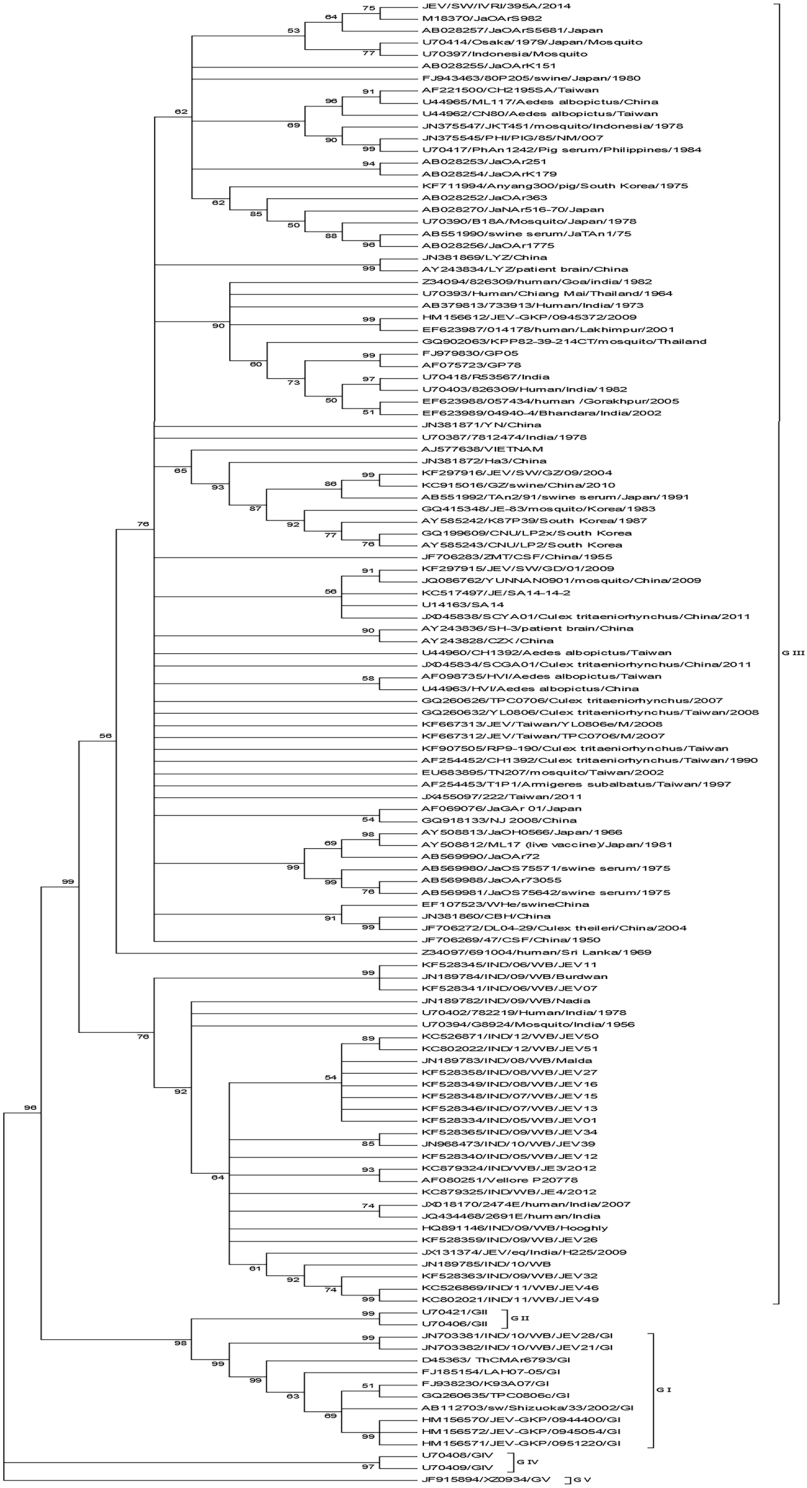
Phylogenetic analysis for the E gene of JEV. The tree was constructed with MEGA6 software using neighbor-joining method based on the kimura-2 parameter model. Data are based on a bootstrap value of 1000 and cutoff value of 50.

Phylogenetic analysis based on E gene of IVRI395A showed that it belonged to genotype III. Maximum identity of 99.9% was seen with Japanese isolate JaOArS982 and 98% with GP78 from India. Viruses closely related to the present swine isolate from India have been detected in humans, mosquitoes and pigs in JEV endemic areas of Asia (Fig 2). The Indian GIII isolates grouped into two distinct clades, Clade-I with GP78 as the prototype virus and Clade-II with Vellore-P20778 as the prototype virus (Fig 2). IVRI395A grouped with Clade-I viruses.

When the partial E gene sequences of the virus strain pertaining to the 2005 outbreak in Uttar Pradesh, India, was compared to the corresponding fragment of the Indian swine isolate, only one nucleotide substitution was noticed. Similar identity was shared by JaOArS982, JaOArS5681 and Osaka/1979 from Japan and JKT220507 from Indonesia.

**Fig 3:**
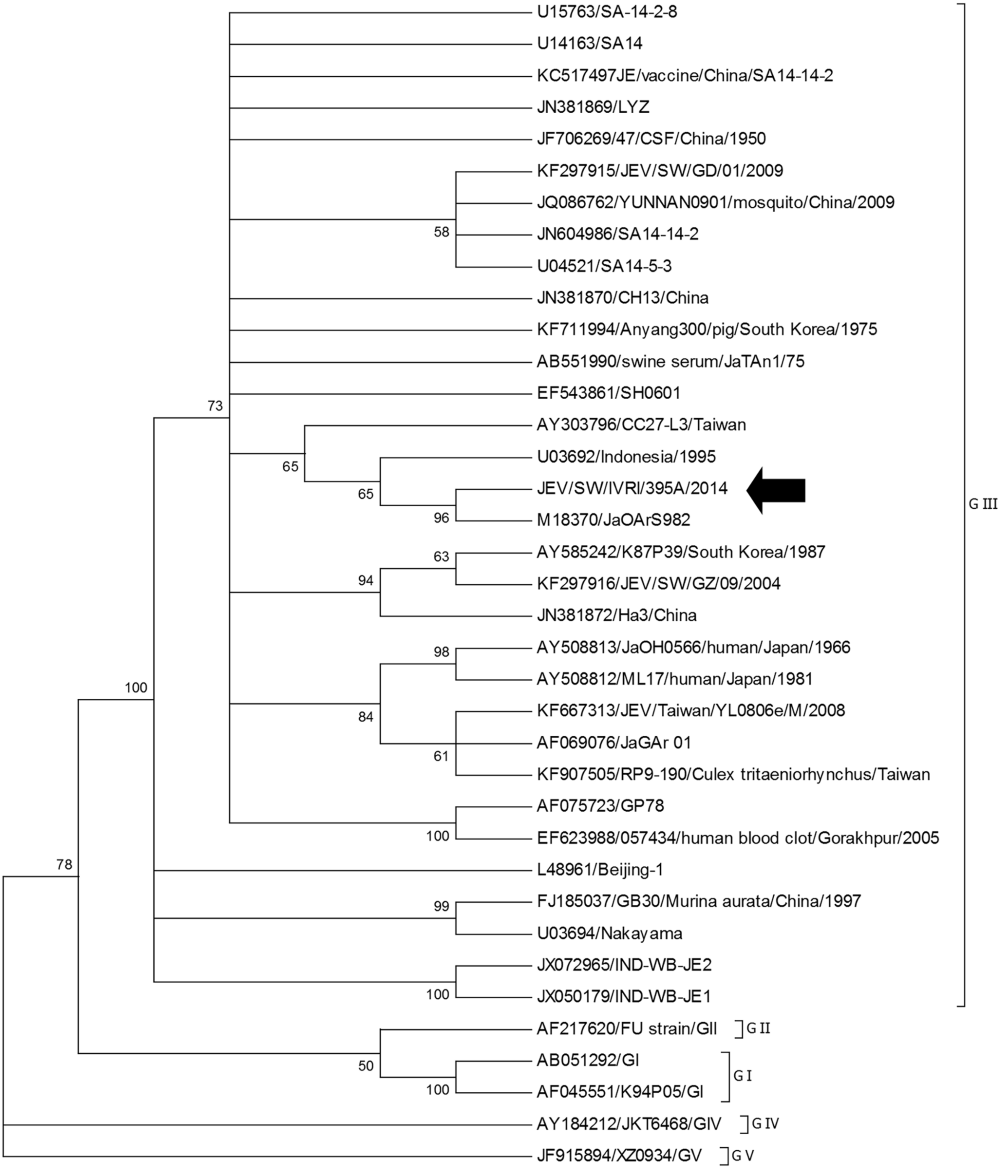
Phylogenetic analysis for the prM gene of JEV. The tree was constructed with MEGA6 software using neighbor-joining method based on the kimura-2 parameter model. Data are based on a bootstrap value of 1000 and cutoff value of 50.

**Fig 4:**
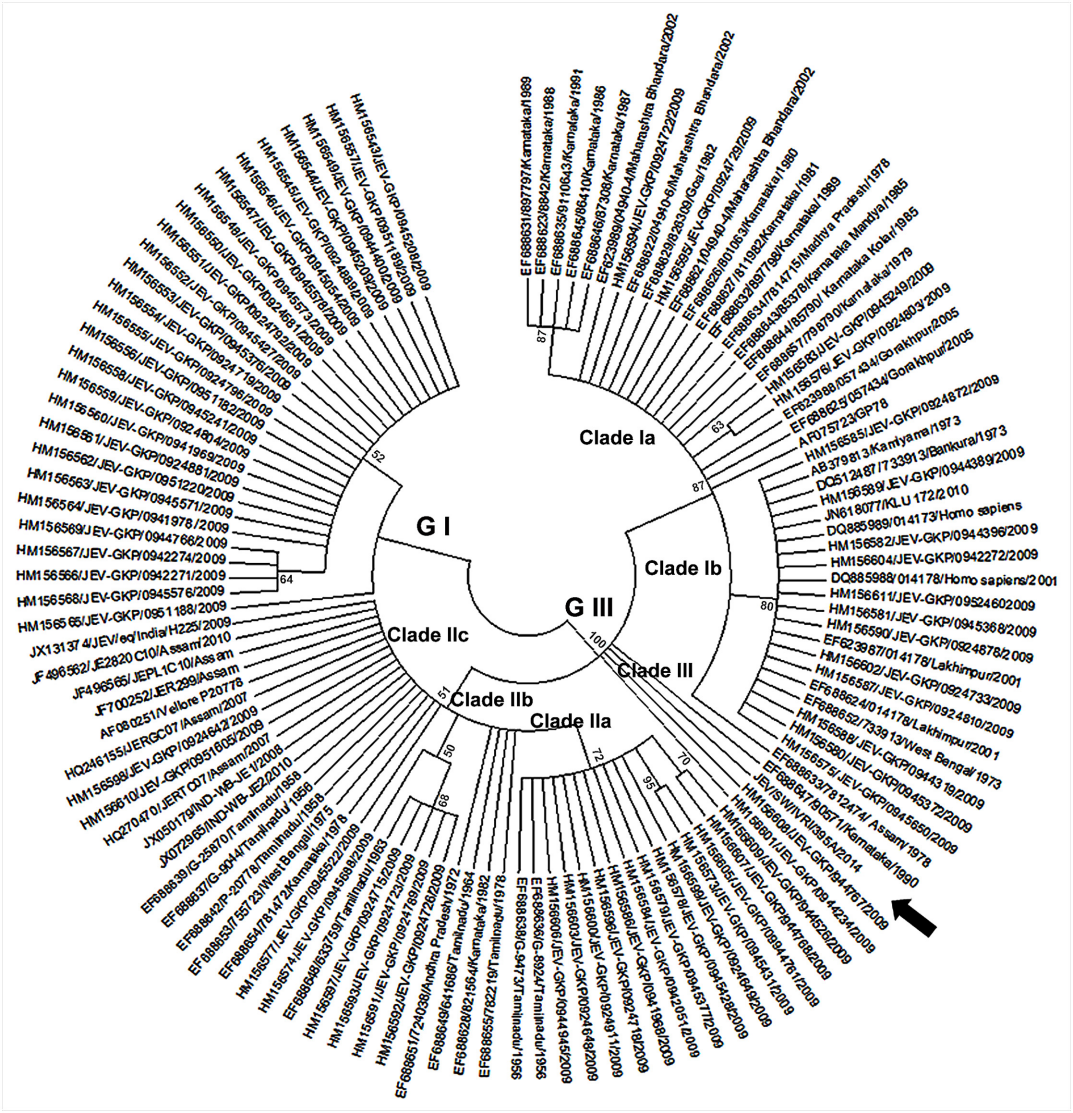
Phylogenetic analysis for prM gene of JEV including sequences data from Indian JEV isolates only. The tree was constructed with MEGA6 software using neighbor-joining method based on the kimura-2 parameter model. Data are based on a bootstrap value of 1000 and cutoff value of 50.

Phylogenetic analysis of the prM gene also showed that IVRI395A belonged to genotype III (Fig 3). Maximum identity of 99.6% was seen with Japanese strain JaOArS982and 96% with IND-WB-JE2 (JX072965) and IND-WB-JE1 (JX050179) from West Bengal, India. The phylogenetic tree constructed with prM gene sequences from India separated into three distinct clades, clade-I, clade-II and clade-III. At the nucleotide level, 5% divergence was noticed between the clade I and clade-II, and their spatial distribution was not restricted to particular regions in the country. The IVRI395A belonged to Clade-III, as were two other isolates (HM156608/944767 and HM156601/944234) collected in 2009 from Gorakhpur, Uttar Pradesh, India. Further it was noted that each of the clade I and clade II could be further subdivided into different subclades: Ia (AF075723/GP78 from Uttar Pradesh as the prototype virus), Ib (EF688652/733913 from West Bengal as the prototype virus), IIa (EF688636/G-8924 from Tamil Nadu as the prototype virus), IIb (EF688648/633759 from Tamil Nadu as the prototype virus), IIc (AF080251/Vellore P20778 from Tamil Nadu as the prototype virus), and clade III (EF688647/90571 from Karnataka as the prototype virus) (Fig 4). Among the various clades and subclades of GIII viruses circulating in India, clade Ia (6 States) and clade IIc (7 States) are more widespread in geographical distribution (Table 4).

**Table 4:** Distribution of various clades of JEV Genotype III in different States of India (1956–2014). Genotype III was further classified into three clades and distribution of different subclades in different states of India, year of detection and number of sequences (in parenthesis) are presented.

Clade	State	Year of Isolation
Ia	Assam	1978(1)
	Madhya Pradesh	1978(1)
	Uttar Pradesh	1978(1), (2005(2), 2009(2)
	Karnataka	1979(1), 1980(1), 1981(1), 1985(2), 1986(1), 1987(1), 1988(1), 1989(2), 1991(1)
	Goa	1982(1)
	Maharashtra	2002(3)
Ib	West Bengal	1973(2)
	Uttar Pradesh	2001(2), 2009(12)
IIa	Tamil Nadu	1956(2)
	Uttar Pradesh	2009(12)
IIb	Tamil Nadu	1963(1)
	Uttar Pradesh	2009(5)
IIc	Tamil Nadu	1956(1), 1958(2), 1964(1), 1978(1)
	Andhra Pradesh	1972(1)
	West Bengal	1975(1), 2008(1), 2010(1)
	Karnataka	1978(1)
	Assam	2007(2)
	Haryana	2009(1)
	Uttar Pradesh	2009(4)
III	Karnataka	1990(1)
	Uttar Pradesh	2009(2), 2014(swine)

## Discussion

In India, JE is an endemic disease particularly in States where rice cultivation is done extensively and pig rearing is practiced by poor, landless farmers with limited resources [22]. Pigs which are known to be amplifiers of JEV should be actively monitored in the country. Moreover pigs infected with JEV may suffer from reproductive problems and this has an adverse effect on profitability of pig farming. But most studies on JEV infection in pigs have been limited to serological surveys in JE endemic areas [15–16] and to the best of our knowledge none have focused on reproductive failure in pigs and genetic characterization of JEV circulating in the Indian pig population.

Investigation of reproductive failure manifested in an increased number of stillborn piglets with neuropathological lesions led to the detection and isolation of JEV as the causative agent. JEV related reproductive failure and stillbirth was first recorded in pigs in Japan (1947–1948), and subsequently JEV was isolated from stillborn piglets [23–24]. JEV can cause stillbirth, mummification, embryonic death and abortion in sows and aspermia in boars [25–28]. In the present study, naturally infected stillborn piglets showed brain lesions as reported in experimentally infected pigs [29].

Presence of serine (S) in E at position 227 in the present swine isolate corroborates earlier observations of amino acids critical for adaptation to Vero cells. It has been reported that amino acid substitution S227P in E can slightly impair the growth rate in Vero cells [20]. The current swine isolate which grows easily in Vero cells may facilitate the development of an inactivated JE vaccine for use in pigs.

Mice inoculated with brain suspensions prepared from stillborn piglets resulted in characteristic neuropathological symptoms and molecular analysis of E identified critical amino acid residues attributable for neurovirulence of JEV [10]. Comparison of E of the current swine isolate with SA14-14-2 (vaccine strain) revealed 10 amino acid substitutions out of which 8 have been reported to be critical for neurovirulence. E-244 has been linked to neurovirulence [9], but the significance of amino acid substitution at E-169 is not known. Interestingly, IVRI395A had a E169I substitution in E as in JaOArS982 isolated in Japan, while all other Indian isolates analyzed in this study had E169V. Further studies are required to ascertain whether this substitution has any significance for virus replication in pigs. In the present study, analysis of the envelope protein of JEV revealed that 5 different haplotypes are circulating in India. While 4 of them have been reported earlier [20] the SKTS haplotype identified in this study pertains to JEV detected in a human case from Chandigarh, India, in 2007. The RKSS haplotype has been associated with positive selection in mosquitoes [20] but our analysis showed that in one instance, this haplotype was detected from a human case of JE in West Bengal, India in 2011 This study revealed that the SKSS haplotype is the predominant haplotype of GIII and GI viruses circulating in humans and mosquitoes in India, and the current Indian swine isolate also belongs to this haplotype.

Phylogenetic analysis based on complete E gene sequences indicated that IVRI395A belonged to genotype III and is closely related to certain isolates from Japan. However, a close analysis of the partial E gene sequences available for the 2005 outbreak in humans in Uttar Pradesh, India, suggested high identity with the present swine isolate. Further, viruses sharing similar identity were detected in 1979 from mosquitoes and humans in Japan and from mosquitoes in Indonesia. This suggested that the present swine isolate is closely related to JEV strains detected in the Asian continent at least three decades ago.

In India, GIII continues to be the predominant genotype [12] but incursion of GI viruses has also been reported [12–13]. Detailed analysis of prM gene sequences of Indian JEV collected from 1956 to 2014 categorized the GIII viruses into three clades. While the clade II viruses were confined to Southern India until 1975, the clade I viruses were mostly detected in North India except for one south Indian State, Karnataka, where it was first detected in 1979 and repeatedly thereafter until 1991. The clade III contained the present Indian swine isolate and three other Indian viruses detected in humans; two from Gorakhpur in North India (2009) and one from Karnataka in the South (1990). This indicates that similar clade III viruses are circulating in human populations in geographically distant locations.

The detection of clade IIc in south India preceded clade Ia in North India. Clade IIc consists of some of the oldest strains (Vellore, P20778 and similar viruses) detected in the South Indian States such as Tamil Nadu, Andhra Pradesh and Karnataka between 1956 and 1978. The clade IIc viruses were first detected in North India in the State of West Bengal (1975) and subsequently in Assam, Haryana and Uttar Pradesh between 2007 and 2009. Clade Ia viruses (GP78 and similar viruses) were first detected simultaneously during 1978 in the North India in the States of Uttar Pradesh, Madhya Pradesh and Assam. Soon after, in 1979, clade Ia viruses were detected in the South India, in Karnataka, where they continued to be detected intermittently until 1991. The 2009 outbreak of JE in the Northern State of Uttar Pradesh showed co-circulation of all three clades of GIII viruses and GI viruses for the first time in India. This suggests that some poorly understood epidemiological factors were responsible for the change in spatial distribution of different genotypes and their clades in India in recent years.

In conclusion, JEV associated with reproductive failure in Indian pigs caused severe neuropathological lesions in stillborn piglets, and the isolated virus belonged to the predominant haplotype of GIII circulating in humans and mosquitoes in India. Analysis of the E protein of JEV and neuropathology in experimentally infected mice characterized IVRI395A as neurovirulent strain. It was phylogenetically related to JEV associated with the 2005 outbreak in India. The spatial distribution of different genotypes and their clades in India has changed in recent years.
